# Supplementary material for: Estimating enteric fever seroincidence in rural western Cambodia: findings from a population-based cross-sectional serosurvey
Source: medRxiv. 2026 Feb 6:2026.02.01.26343908. Preprint. [Version 1] doi: 10.64898/2026.02.01.26343908 (PMC12889769; doi:10.64898/2026.02.01.26343908)
Supplement: Supplement 1 [file media-1.pdf]

Supplementary materials

Table 1. Seroincidence estimated by IgA or IgG alone, overall, by age group and district.

Characteristics	Overall*	Koas Krala*	Pailin*	Rukhakiri*	Samlout*
**IgA**					
Overall	156.5 (132.2, 185.3)	154.4 (105.2, 226.7)	174.5 (130.2, 233.9)	134.8 (83.5, 217.5)	152.3 (116.7, 198.8)
5-15 years	167.6 (138.7, 202.6)	178 (110, 288.2)	153.9 (117.3, 201.8)	132.5 (76.4, 229.9)	182.7 (137.2, 243.3)
16-25 years	127.7 (97, 168.3)	99.8 (68.2, 146.1)	275.9 (110.1, 691.8)	144.6 (68.8, 304.1)	97.4 (71.2, 133.4)
**IgG**					
Overall	165.2 (145.8, 187.2)	257 (201, 328.6)	149.8 (126.5, 177.4)	180.4 (153.8, 211.6)	132.5 (107.8, 162.9)
5-15 years	157.7 (135.7, 183.3)	258.7 (194, 345)	134.2 (111.7, 161.3)	194.7 (163.7, 231.6)	120.6 (92.7, 156.7)
16-25 years	189.8 (162, 222.5)	251 (193.3, 326.1)	201.7 (143.9, 282.7)	135.3 (86, 212.7)	168.1 (136.1, 207.7)
*Seroincidence (95%CI), all reported per 1,000 person-years					

Table 2. Comparisons of enteric fever seroincidence using two-sample z-test, by district, age group, WASH access, household wealth status, and month of data collection.

Variable	Comparison group	Seroincidence differences (95%CI)*	z statistic	p-value
**District**				
	Koas Krala vs.Samlout	69.6 (6.5, 132.7)	2.1619354	0.030623
	Koas Krala vs.Pailin	50.9 (-13, 114.7)	1.5619949	0.118289
	Koas Krala vs.Rukhakiri	47.9 (-22, 117.8)	1.3427298	0.17936
	Pailin vs.Rukhakiri	-3 (-50.1, 44.1)	-0.1242687	0.901102
	Pailin vs.Samlout	18.7 (-17.5, 54.9)	1.0141354	0.310518
	Rukhakiri vs.Samlout	21.7 (-24.4, 67.8)	0.9238461	0.355566
**Age group**				
	16-25 years vs. 5-15 years	-18.5 (-53.1, 16.1)	-1.0494292	0.293981
Age group by district				
Koas Krala	16-25 years vs. 5-15 years	-80.9 (-173.7, 11.8)	-1.7109965	0.087082
Rukhakiri	16-25 years vs. 5-15 years	-45.5 (-131.9, 40.9)	-1.032052	0.302048
Pailin	16-25 years vs. 5-15 years	55.6 (-35.3, 146.5)	1.1995785	0.230303
Samlout	16-25 years vs. 5-15 years	-22.9 (-64.7, 18.9)	-1.0732852	0.283143
**WASH access**				
	≥1 unimproved vs. Both improved	1.8 (-33.6, 37.2)	0.099512924	0.920731
WASH access by district				

Koas Krala	≥1 unimproved vs. Both improved	19.6 (-101.6, 140.8)	0.317213171	0.751082
Rukhakiri	≥1 unimproved vs. Both improved	3.8 (-60.3, 67.9)	0.117164165	0.90673
Pailin	≥1 unimproved vs. Both improved	-1.3 (-61.6, 58.9)	-0.043359932	0.965415
Samlout	≥1 unimproved vs. Both improved	-25.3 (-71.6, 21)	-1.071436529	0.283973
WASH access by age group				
16-25 years	≥1 unimproved vs. Both improved	-39.5 (-98.4, 19.5)	-1.312910832	0.189213
5-15 years	≥1 unimproved vs. Both improved	15.9 (-24.8, 56.6)	0.766736319	0.443238
**Household wealth status**				
	Poor vs. Middle	19.9 (-24.4, 64.2)	0.88027989	0.378708
	Poor vs. Rich	16.7 (-19.7, 53.1)	0.898276189	0.369038
	Middle vs. Rich	-3.2 (-40.3, 33.8)	-0.170972841	0.864245
Household wealth status by district				
Koas Krala	Poor vs. Middle	-0.3 (-136.9, 136.2)	-0.004530605	0.996385
	Poor vs. Rich	-3.9 (-94, 86.2)	-0.085042552	0.932228
	Middle vs. Rich	-3.6 (-144.8, 137.6)	-0.049876798	0.960221
Rukhakiri	Poor vs. Middle	-5.5 (-91.2, 80.3)	-0.124873789	0.900623
	Poor vs. Rich	48.4 (-23.8, 120.6)	1.314130348	0.188802
	Middle vs. Rich	53.9 (-3.3, 111.1)	1.845607444	0.064949
Pailin	Poor vs. Middle	69.4 (-12.4, 151.3)	1.663513065	0.09621
	Poor vs. Rich	59.4 (-13.7, 132.6)	1.59269191	0.111229
	Middle vs. Rich	-10 (-61.4, 41.4)	-0.381721413	0.702668
Samlout	Poor vs. Middle	-25.6 (-76.3, 25.2)	-0.987813232	0.323244
	Poor vs. Rich	-33.8 (-72.6, 5)	-1.709325066	0.087391
	Middle vs. Rich	-8.2 (-60.7, 44.2)	-0.307196074	0.758694
Household wealth status by age group				
16-25 years	Poor vs. Middle	-10.7 (-67.5, 46.1)	-0.369037443	0.7121
	Poor vs. Rich	-32.9 (-89.3, 23.6)	-1.141076156	0.253838
	Middle vs. Rich	-22.2 (-81.8, 37.4)	-0.729079617	0.465953
5-15 years	Poor vs. Middle	27.7 (-25.6, 81)	1.020037808	0.307711
	Poor vs. Rich	31.1 (-11.5, 73.6)	1.429762508	0.152785
	Middle vs. Rich	3.3 (-41.1, 47.8)	0.146484589	0.883539
**Month of data collection**				
	Apr, May vs. Aug	-180.5 (-284.1, -76.9)	-3.414646782	0.000639
	Apr, May vs. Jun	39.4 (5.7, 73.1)	2.293753299	0.021805
	Apr, May vs. Oct, Nov	0.6 (-39.1, 40.4)	0.030594313	0.975593
	Apr, May vs. Sep	2.1 (-31.9, 36.1)	0.12044058	0.904134

	Aug vs. Jun	219.9 (116.9, 322.9)	4.183158951	2.87E-05
	Aug vs. Oct, Nov	181.1 (75.9, 286.3)	3.374805416	0.000739
	Aug vs. Sep	182.6 (79.4, 285.7)	3.46991203	0.000521
	Jun vs. Oct, Nov	-38.8 (-77.1, -0.5)	-1.986599951	0.046967
	Jun vs. Sep	-37.3 (-69.5, -5.1)	-2.2719975	0.023087
	Oct, Nov vs. Sep	1.5 (-37.1, 40)	0.074601211	0.940532
*Seroincidence differences (95%CI) reported per 1,000 person-years				

Table 3. HLyE IgA and IgG seroincidence by month of data collection

**Data collection period ***	**Number of participants**	**Seroincidence (95%CI) / 1,000 person-years^**
Apr, May†	137	150.7 (127.6, 177.9)
Jun	70	111.2 (90.8, 136.2)
Aug	96	331.1 (244.4, 448.6)
Sep	118	148.6 (127.3, 173.4)
Oct, Nov†	108	150.0 (122.1, 184.4)

* Sample collection occurred during 8 months of the recruitment period (all activities paused in July due to local political activities, and no samples were collected in December). The data collection months of each district are: Rukhakiri- April (n=46), May (n=8); Koas Krala- August (n=67), September (n=69); Samlout- May (n=83), June (n=70), August (n=29), November (n=16); Pailin- September (n=49), October (n=92).

† Data from April and May were combined, as were the months October and November, due to the small number of participants recruited in April and November.

^Seroincidence differed significantly by month of data collection, with August showing higher estimates than all other months (p<0·001), and June differing significantly from April, May, September, and October, November (p<0·05).

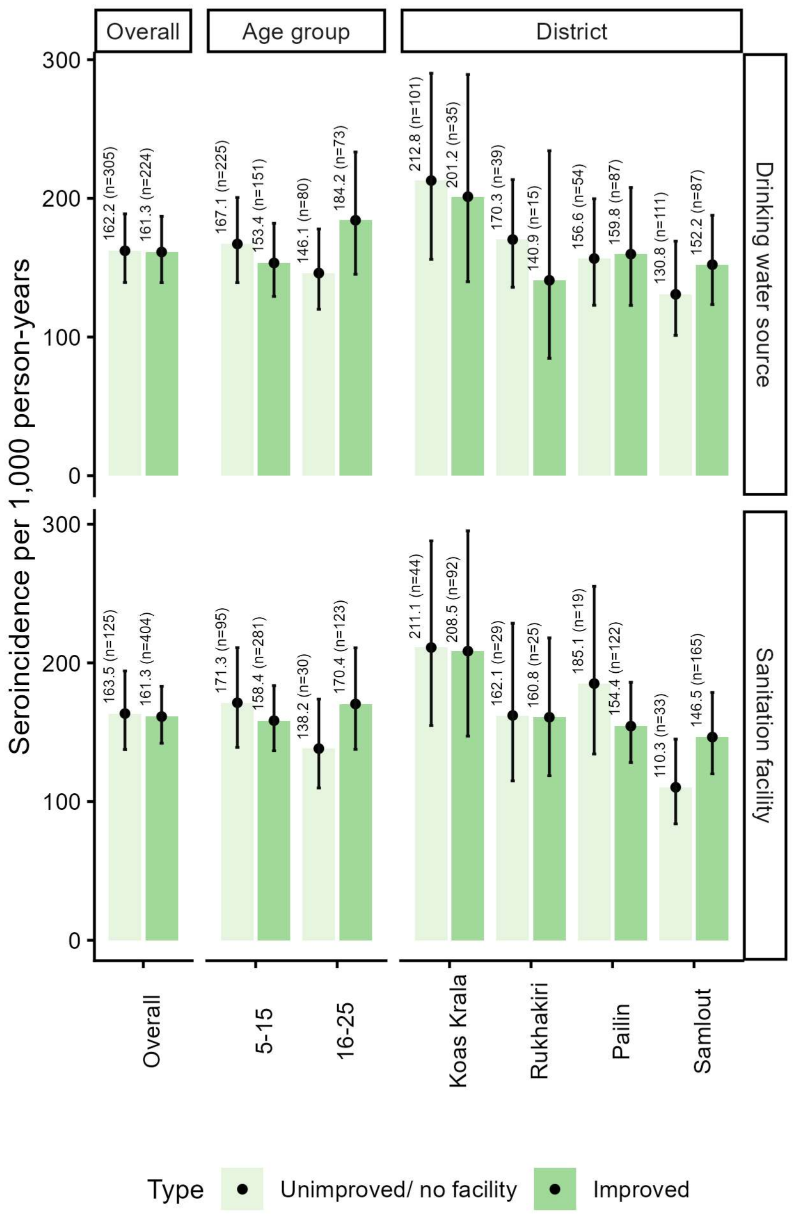

Figure 1. Enteric fever seroincidence estimates based on quantitative antibody responses to HLyE IgA and IgG, by drinking water source (top) and sanitation facility type (bottom), overall and stratified by age and district.

No statistically significant differences in seroincidence were observed between improved and unimproved drinking water sources or sanitation facilities across age groups, districts, or overall.

For sanitation facilities, the “Unimproved” and “No facilities” categories were combined due to small sample sizes. Seroincidence estimates are shown, with the number of observations indicated in parentheses.
